# Solute removal during continuous renal replacement therapy in critically ill patients: convection versus diffusion

**DOI:** 10.1186/cc4903

**Published:** 2006-04-28

**Authors:** Zaccaria Ricci, Claudio Ronco, Alessandra Bachetoni, Giuseppe D'amico, Stefano Rossi, Elisa Alessandri, Monica Rocco, Paolo Pietropaoli

**Affiliations:** 1 Department of Intensive Care, Policlinico Umberto I, Rome, Italy; 2 Department of Nephrology, Dialysis and Transplantation, St Bortolo Hospital, Vicenza, Italy; 3 Laboratory of Clinical Chemistry, Policlinico Umberto I, Rome, Italy; 4 Department of Intensive Care, Policlinico Umberto I, Rome, Italy; 5 Department of Intensive Care, Policlinico Umberto I, Rome, Italy

## Abstract

**Introduction:**

The best modality, for continuous renal replacement therapy (CRRT) is currently uncertain and it is poorly understood how transport of different solutes, whether convective or diffusive, changes over time.

**Methods:**

We conducted a prospective cross over study in a cohort of critically ill patients, comparing small (urea and creatinine) and middle (β_2 _microglobulin) molecular weight solute clearance, filter lifespan and membrane performance over a period of 72 hours, during 15 continuous veno-venous dialysis (CVVHD) and 15 continuous veno-venous hemofiltration (CVVH)sessions. Both modalities were administered based on a prescription of 35 ml/kg/h and using polyacrylonitrile filters.

**Results:**

Median filter lifespan was significantly longer during CVVHD (37 hours, interquartile range (IQR) 19.5 to 72.5) than CVVH (19 hours, IQR 12.5 to 28) (*p *= 0.03). Median urea time weighted average (TWA) clearances were not significantly different during CVVH (31.6 ml/minute, IQR 23.2 to 38.9) and CVVHD (35.7 ml/minute, IQR 30.1 to 41.5) (*p *= 0.213). Similar results were found for creatinine: 38.1 ml/minute, IQR 28.5 to 39, and 35.6 ml/minute, IQR 26 to 43 (*p *= 0.917), respectively. Median β_2_m TWA clearance was higher during convective (16.3 ml/minute, IQR 10.9 to 23) than diffusive (6.27 ml/minute, IQR 1.6 to 14.9) therapy; nonetheless this difference did not reach statistical significance (*p *= 0.055). Median TWA adsorptive clearance of β_2_m appeared to have scarce impact on overall solute removal (0.012 ml/minute, IQR -0.09 to 0.1, during hemofiltration versus -0.016 ml/minute, IQR -0.08 to 0.1 during dialysis; *p *= 0.79). Analysis of clearance modification over time did not show significant modifications of urea, creatinine and β_2_m clearance in the first 48 hours during both treatments. In the CVVHD group, the only significant difference was found for β_2_m between 72 hours and baseline clearance.

**Conclusion:**

Polyacrylonitrile filters during continuous hemofiltration and continuous hemodialysis delivered at 35 ml/kg/h are comparable in little and middle size solute removal. CVVHD appears to warrant longer CRRT sessions. The capacity of both modalities for removing such molecules is maintained up to 48 hours.

## Introduction

There has been growing interest in the effects of continuous renal replacement therapy (CRRT) on the course of acute renal failure (ARF) in critically ill patients, based on the assumption that removal of several molecules, including uremic toxins and inflammatory mediators, might improve outcome [1,2]. Different prospective trials have provided conflicting results regarding what dose should be applied in the extracorporeal therapy of ARF [3-5]. Furthermore, there is wide variation in the way in which CRRT is practiced around the world. In addition to dosing, timing, membranes and fluids, the mode of CRRT varies. Many intensivists and nephrologists prefer to use continuous veno-venous hemofiltration (CVVH) in the belief that pure convection will remove more larger molecules than diffusion-based continuous veno-venous dialysis (CVVHD). Others argue that CVVHD is easier and, given the lack of comparative evidence, prefer this mode. Still a third school favors continuous veno-venous hemodiafiltration (CVVHDF) on the basis that without evidence, providing both modes is safest. Many studies have used continuous hemofiltration for this purpose, following the expectation that a wider range of molecular weights can be cleared with predominantly convective rather than predominantly diffusive techniques [3-5]. However, this notion, although based on several *in vitro *experiments and experience in chronic dialysis [6], has never been tested by a comparative study during the course of continuous extracorporeal treatment.

We recently showed that CRRT dose, estimated as urea clearance, is highly predictable, regardless of prescription and selected modality [7]. During CRRT, nonetheless, many variables may affect the effective delivery of treatment dose: if the molecular weight of different solutes is certainly an important aspect, the time factor appears to be an essential variable as well; interruptions cause a clinically significant therapy downtime and increase discrepancy between prescription and effective delivery [8]. A recent single center study showed that mean filter life in critically ill patients was only 16 hours and that clotting was the primary reason for shortened filter life [9]. Furthermore, progressive filter clotting and clogging may greatly reduce, over time, filter performance and solute removal. Adsorption is another mechanism of mediator removal for some membranes, particularly for polyacrylonitrile membranes during hemofiltration [10]. We conducted a prospective cross over study in a cohort of critically ill patients, comparing small and middle molecular weight solute clearance, filter lifespan and membrane performance over a period of 72 hours during CVVHD and CVVH. Urea and creatinine were used as markers of small molecular weight solutes and β_2 _microglobulin (β_2_m) as a surrogate for middle molecular weight solutes.

## Materials and methods

We prospectively collected data from 30 consecutive CRRT treatments administered to 15 critically ill patients (Table 1) with ARF. The study protocol was approved by the local ethics committee. CRRT was started in each case when a 'Failure' level was indicated according to RIFLE criteria. RIFLE is an acronym indicating Risk of renal dysfunction; Injury to the kidney; Failure of kidney function, Loss of kidney function and End-stage kidney disease [11]. Thus, criteria were a three-fold increase of creatinine level with respect to baseline, or a creatinine concentration over 4 mg/dl, or oligoanuria lasting for 24 hours.

According to the mean of a previously described 'Calculator', CRRT was prescribed for both CVVHD and CVVH to a dose of single pool Kt/V (clearance (K) × time (t)/urea volume of distribution (V)) of 1.4, which approaches an operative prescription of 35 ml/kg/h [7]. Lactate buffered solutions were used. The replacement solution during CVVH was infused both before and after the filter to keep the plasmatic filtration fraction below 20%. Net ultrafiltration (UF) was determined based on clinical needs.

Anticoagulation was performed, according to protocol, with an unfractionated pre-filter heparin infusion at a dose of 5 to 10 IU/kg/h. No anticoagulant infusion was administered if one of the following was present: ongoing bleeding; major hemorrage in the last seven days; an international normalized ratio (INR) or a partial thromboplastin time ratio (PTTr) higher than 2; or a platelet count lower than 40 × 10^3^/mm^3^. Treatments were interrupted only at filter clotting (filter drop pressure over 300 mmHg) or clogging (transmembrane pressure over 350 mmHg).

A Prismaflex machine (Gambro-Dasco, Mirandola (Mo) Italy) and 0.9 m^2 ^AN69 hollow fibre filters (Multiflow 100, Hospal, Lyon, France) were used for all treatments (UF coefficient with blood, 25 ml/h/mmHg × m^2^; cutoff point, 40 kDa). Only the first two treatments for each patient were considered for the study; after the criteria for CRRT were established, patients received one CVVH and one CVVHD session, in a random sequence. The cross over design of the study allowed us to compare convective and diffusive clearances and to avoid eventual confounding factors deriving from different vascular access, anticoagulation requirements, net UF needs or other unknown elements (Tables 1 and 2).

Samples were withdrawn at treatment start (T0), after 12 hours (T1) and then every 24 hours (T2, T3, T4). One sample withdrawn at the 72nd hour and three at the 96th hour of CVVH and one at the 120th hour of CVVHD were excluded from the analysis of clearance modification over time.

Samples for urea, creatinine and β_2_m measurement were taken simultaneously from the arterial blood line (before the pre-filter re-infusion site during CVVH), from the venous blood line (after the post-filter re-infusion line during CVVH) and from the effluent line (df). Blood samples were then centrifuged and stored at -80°C together with effluent samples. Quantitative measures of β_2_m were obtained by microparticle enzyme immunoassay.

Urea, creatinine and β_2_m clearances were calculated from the dialysate side using the formula:

K = 2 × ([X]_df _× Q_df_)/([X]_I _+ [X]_O_)

where [X]_df _is the concentration of a given solute X in the diafiltrate; [X]_I _is the concentration of the examined solute X in the plasma entering the filter; [X]_O _is the plasma concentration of the examined solute X in the venous blood line; and Q_df _is the diafiltrate (effluent) flow rate (ml/minute).

Adsorptive clearance (K_AD_) was calculated only for β_2_m as:

K_AD _= M_AD_/[X]_I_

where M_AD _is the mass removal rate by membrane adsorption, which was calculated as:

M_AD _= M_t _- M_df_

where M_t _is total mass removal rate (pg/minute):

M_t _= M_I _- M_O_

M_df _is mass removal rate by UF or dialysis:

M_df _= Q_df _× [X]_df_

M_I _is inlet mass rate (mg/minute):

M_I _= Q_I _× [X]_I_

M_O _is outlet mass rate (pg/minute):

M_O _= Q_O _× [X]_O_

Q_I _is inlet plasma flow (ml/minute):

Q_I _= Q_B_(1 - hematocrit)

Q_O _is outlet plasma flow (ml/minute):

Q_O _= Q_I _- Q_df_

and Q_df _is the effluent flow (ml/minute) and Q_B _is prescribed blood flow (ml/minute).

### Statistical analysis

Statistical analysis was performed with the GraphPad Prism 4.03 software package (GraphPad Software, San Diego, CA, USA). Data are reported as median ± interquartile range (IQR). Comparative circuit survival for each modality was assessed using log-rank tests with Kaplan-Meier analysis. Fisher exact test and Mann-Whitney tests when appropriate were used to compare clinical features of the patients and prescribed settings during CVVH and CVVHD. Time-weighted average (TWA) clearances (excluding the baseline value) were calculated for the two techniques and used for comparing CVVH and CVVHD by Mann-Whitney test. To evaluate filter performance over time, clearances of each solute were then analyzed separately for CVVH and CVVHD by Friedman's test. *Post hoc *Dunn tests were used to identify which time points were associated with change. A *p *value less than 0.05 was considered significant.

## Results

We examined 30 treatments (15 CVVH and 15 CVVHD) administered to 15 consecutive critically ill patients. Clinical characteristics of the patients and circuit pressures at the moment the session was started were compared (Tables 1 and 2): no significant differences were observed for patient weight, age, severity of illness scores (Simplified Acute Physiology Score II), anticoagulation parameters (international normalized ratio, partial thromboplastin time ratio, Antithrombin III, heparin infusion prescription, net UF requirements, blood flow rate prescription, vascular access conditions (cannulation site, internal diameter, access and return pressure) and reasons to stop treatment (clogging/clotting).

**Figure 1:**
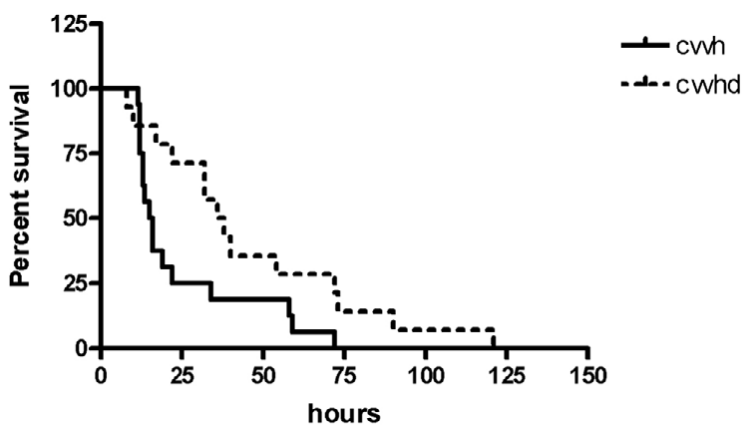
Kaplan-Meier analysis of circuit survival for continuous veno-venous hemofiltration (CVVH) and continuous veno-venous dialysis (CVVHD).

**Figure 2:**
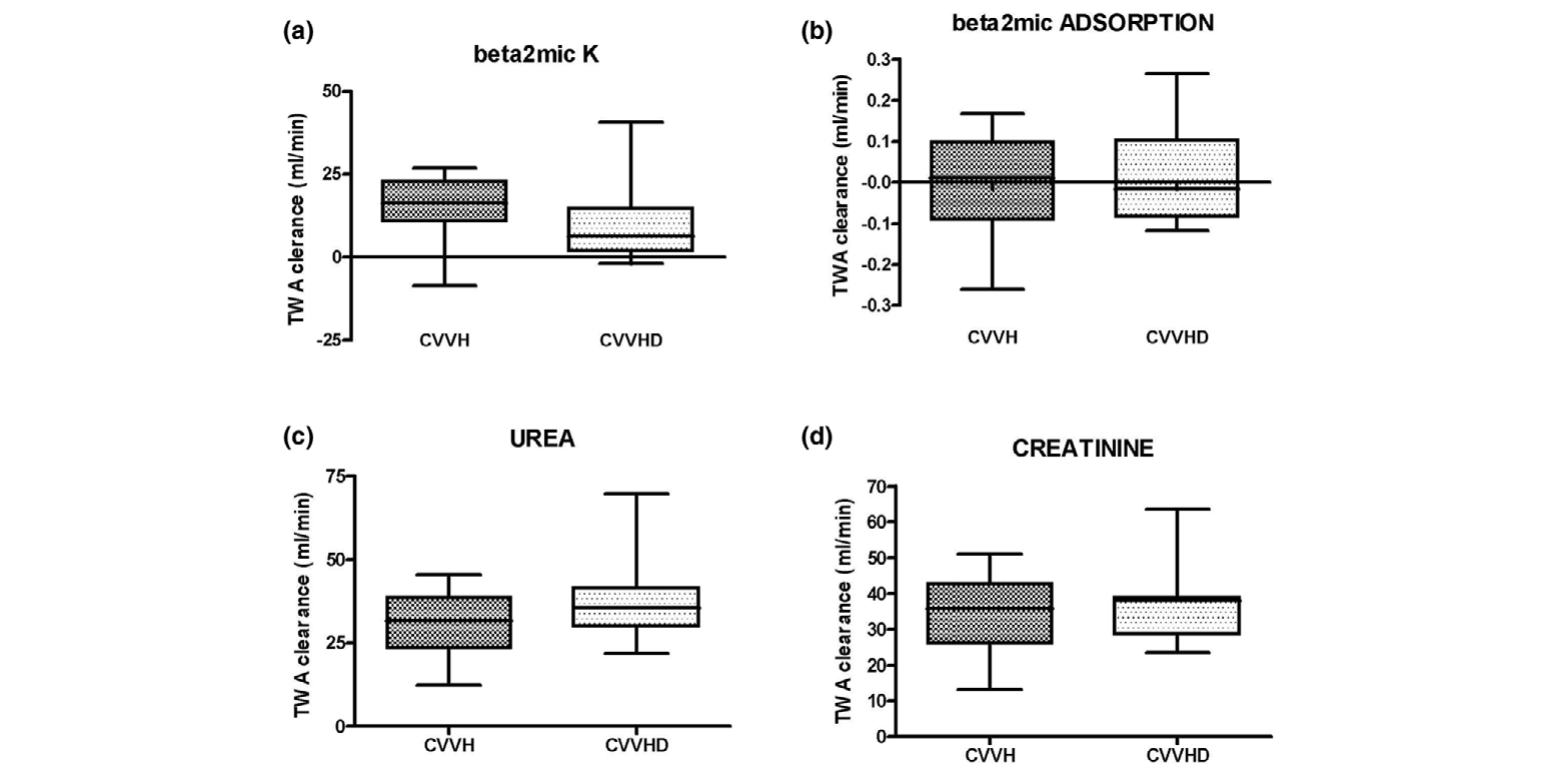
Time weighted average (TWA) clearance of **(a) **β_2 _microglobulin (beta2mic), **(c) **urea and **(d) **creatinine by convective and diffusive transport. **(b) **Beta2mic adsorptive clearance during continuous veno-venous hemofiltration (CVVH) and continuous veno-venous dialysis (CVVHD). Data are expressed as median and interquartile range. None of these comparisons reaches statistical significance.

Median filter lifespan was significantly longer during CVVHD (37 hours, IQR 19.5 to 72.5) than CVVH (19 hours, IQR 12.5 to 28) (*p *= 0.03; Figure 1). Median urea TWA clearances were not significantly different between CVVH (31.6 ml/minute, IQR 23.2 to 38.9) and CVVHD (35.7 ml/minute, IQR 30.1 to 41.5) (*p *= 0.213). Similar results were found for creatinine: 38.1 ml/minute, IQR 28.5 to 39, and 35.6 ml/minute, IQR 26 to 43 (*p *= 0.917), respectively. Median β_2_m TWA clearance was higher during convective (16.3 ml/minute, IQR 10.9 to 23) than diffusive (6.27 ml/minute, IQR 1.6 to 14.9) therapy; however, this difference was not statistically significant (*p *= 0.055). Median TWA adsorptive clearance of β_2_m appeared to be superior during hemofiltration (0.012 ml/minute, IQR -0.09 to 0.1, versus -0.016 ml/minute, IQR -0.08 to 0.1, during dialysis), but again without significant differences (*p *= 0.79; Figure 2a–d).

**Figure 3:**
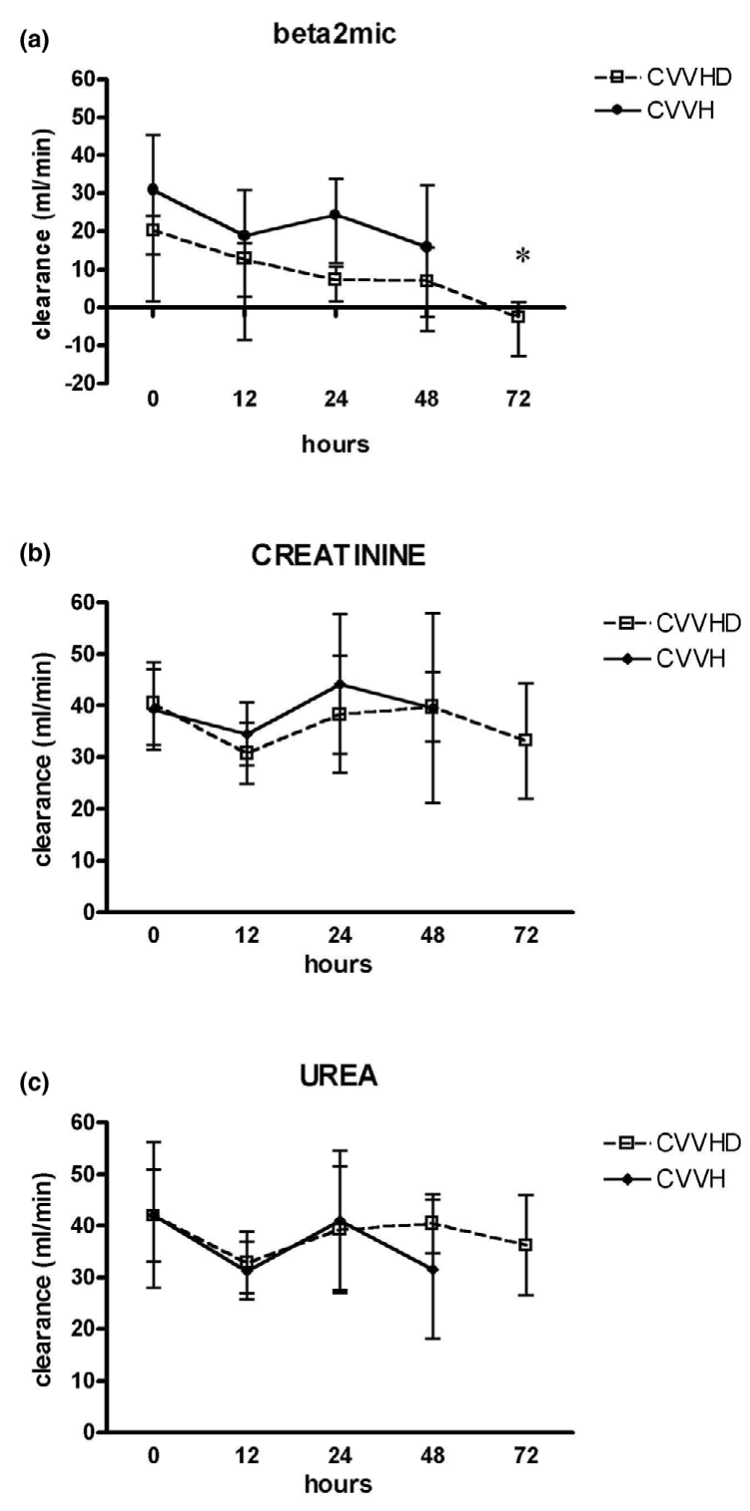
Behaviour of **(a) **β_2 _microglobulin (beta2mic), **(b) **creatinine and **(c) **urea clearance over time for continuous veno-venous hemofiltration (CVVH) and continuous veno-venous dialysis (CVVHD). Beta2mic removal decreased significantly with respect to baseline during CVVHD at T4 (72 hours). Data are expressed as median and interquartile range. The asterisk indicates *p *< 0.05.

Analysis of clearance modification over time was performed separately for predominantly convective and predominantly diffusive treatments. During CVVH, available data did not show significant modifications of urea, creatinine and β_2_m clearance in the first 48 hours. During CVVHD, the only significant difference was found between the T4 (72 hours) and baseline clearance of β_2_m. Results for each solute are shown in Figure 3a–c.

## Discussion

During CRRT, the ideal membrane should remove the widest range of waste products and inflammation mediators peaking in the blood of anuric septic patients. At the start of treatment, when the filter is fresh, hemofiltration is probably the modality that provides, through convective transport, the maximum solute removal allowed by the filter pore cutoff. It has never been evaluated, however, whether filter performance is maintained over time during CRRT. Furthermore, recent preliminary studies have questioned whether, when using polyacrylonitrile or high porosity membranes, hemofiltration is superior to diffusion at eliminating high molecular weight molecules [12-14].

Our study aimed to give some answers to these issues by comparing the clearance of small (urea and creatinine) and middle (β_2_m) size molecules during the course of CVVH and CVVHD. Urea and creatinine are the classic serum markers measured during ARF to monitor the progression of the disease or the efficacy of extracorporeal treatment. β_2_m is an 11,600 Dalton molecule that is normally present in most biological fluids; it is filtered by glomeruli and is catabolised after proximal tubular reabsorption. Serum β_2_m levels are elevated up to 60-fold in patients undergoing dialysis for chronic renal failure and β_2_m amyloid deposits are responsible for several musculoskeletal manifestations in these patients [15]. The role of this substance during ARF is unknown, but we used it as a marker for middle molecular weight molecules (such as, endothelin, bradykinin, IL1, IL8, complement factors C3a/C5a) that range between 10 and 50 kDa.

During the study we used polyacrylonitrile filters, which are biocompatible synthetic membranes with both a high ultrafiltration coefficient (K_uf_) and a very good diffusive solute transfer: these filters have a relatively high cutoff point and are also considered to have good adsorptive properties [10]. The prescribed dose for both modalities was a Kt/V of 1.4, which approaches a urea clearance of 35 ml/kg/h. This dose was recently described to improve the outcome of a large population of critically ill patients, if administered by CVVH, and was suggested by the Surviving Sepsis Campaign guidelines as the recommended prescription during septic ARF [3,16].

During CVVH, we delivered re-infusion solution partially before and partially after the filter: the rate of pre-/post-dilution was calculated after the blood flow was set in order to keep the plasmatic filtration fraction below 20%. However, considering that the median weight of patients was 75 kg and that the median pump blood flow was 150 ml/minute during CVVH, high UF rates were always necessary, even using predilution. This condition caused significantly higher transmembrane pressure values during CVVH than during CVVHD (data not shown), which could partially explain the fact that continuous dialysis sessions were correlated with a longer filter lifespan than in hemofiltration.

As expected, the clearance of small molecules was very similar between the two groups and this confirmed that the operative prescription was correct and comparable. Elimination of β_2_m was superior during hemofiltration even if, after adjustment for the time weight average, the difference was not statistically significant. Although we can speculate that with a larger sample size we might have achieved a significant *p *value, we must remark that the higher degree of continuity allowed by diffusive treatments and the consequent lower downtime, when reported for two independent populations, might further balance this difference in the clearance of middle molecular weight molecules. Moreover, irregardless of which modality was used, the performance of the membranes in removing small and middle molecular weight solutes was good up to 48 hours.

In light of these results, if a 35 ml/kg/h dose is prescribed during a continuous treatment with polyacrylonitrile membranes, hemofiltration and hemodialysis seem to be equipotent at clearing low and middle molecular weight solutes. During dialysis, we noticed a significant reduction in β_2_m clearance at the 72nd hour. Our strict anticoagulation protocol was designed to infuse as little heparin as possible, aiming to reduce bleeding events. When examined in the light of new anticoagulation strategies that report filter lifespans of over 100 hours [17], filter performance could probably be prolonged beyond the limit of 48 to 72 hours. Based on our results, the impact of adsorption on overall clearance of β_2_m was modest, always being less than 0.01%. Changes in adsorption over time were insignificant.

Some limits of the study must be addressed. The sample size was limited to only 15 patients because it showed a good discriminative power to detect differences in filter lifespan; a bigger sample might cause the difference in β_2_m clearance between CVVH and CVVHD to increase. Secondly, we used only polyacrylonitrile filters because they are one of the most commonly used biosynthetic membranes worldwide: our results can not be extended to other hemodialyzers. Finally, β_2_m was chosen as a surrogate for cytokines and other 10 to 50 kDa molecules because it could be easily measured at our center: however, actual clearance of middle molecular weight mediators, which share a similar molecular weight but different biochemical properties, may differ greatly from β_2_m.

## Conclusion

In a cohort of 15 critically ill patients requiring extracorporeal purification, polyacrylonitrile filters during continuous hemofiltration and continuous hemodialysis delivered at 35 ml/kg/h are comparable for the removal of small and middle molecular weight solutes. CVVHD appears to warrant longer CRRT sessions. The capacity of both modalities for removing such molecules is maintained up to 48 hours.

## Key messages

• 	CVVHD appears to warrant longer CRRT sessions than CVVH.

• 	Polyacrylonitrile filters during CVVH and CVVHD delivered at 35 ml/kg/h are comparable for the removal of small and middle molecular weight solutes.

• 	The capacity of both modalities for removing such molecules is maintained up to 48 hours.

## Abbreviations

β_2_m = β_2 _microglobulin; ARF = acute renal failure; CRRT = continuous renal replacement therapy; CVVH = continuous veno-venous hemofiltration; CVVHD = continuous veno-venous dialysis; IQR = interquartile range; TWA = time weighted average; UF = ultrafiltration.

## Competing interests

The authors declare that they have no competing interests.

## Authors' contributions

ZR designed the study, participated in data collection and drafted the paper. CR designed the study and participated in data interpretation. AB participated in laboratory analyses. GD, SR and EA participated in data collection. MR and PP revised the article.

## Figures and Tables

**Table 1:** Clinical characteristics and vascular access features of examined patients

Total number of patients	15
Admission diagnosis	
Abdominal surgery	5
Pneumonia	5
Trauma	4
Acute myocardial infarction	1
SAPS II^a^	61 (15–80)
Age^a^	50 (25–78)
Weight^a^	75 (58–89)
Males/females	10/5
Catheter placement	
Femoral	9
Internal jugular	3
Subclavian	3
Catheter size	
12 French	7
13.5 French	8

^a^Simplified Acute Physiology Score (SAPS) II, age and weight are median with interquartile range in parentheses.

**Table 2:** Coagulation parameters of examined patients and machine settings at the start of each treatment

Parameter	CVVH	CVVHD	P-value
INR	1.21 (0.98–1.95)	1.4 (0.95–2)	NS
PTTr	1.5 (0.85–2.1)	1.19 (0.87–1.89)	NS
PLT count × 1,000	248 (56–434)	231 (65–358)	NS
Antithrombin III (%)	75 (89-56)	69 (94-58)	NS
Hematocrit (%)	25 (21.5–35)	24.7 (22–34.5)	NS
Heparin (U/h)	500 (150–750)	500 (200–800)	NS
Q_B_	150 (110–180)	135 (115–165)	NS
Q_REP _(ml/h)		-	-
Pre	1,450 (800–1,700)		
Post	1,500 (1,000–1,950)		
Q_DIAL _(ml/h)	-	2,150 (1,800–2,800)	-
Net UF	150 (50–350)	165 (45–300)	NS
Access pressure (mmHg)	-75 (-35 to -170)	-82 (-40 to -155)	NS
Return pressure (mmHg)	90 (65–178)	82 (46–160)	NS
Clotting/clogging	9/6	7/8	NS

Data are presented as median with interquartile range in parentheses. CVVH, continuous veno-venous hemofiltration; CVVHD, continuous veno-venous dialysis; INR, international normalized ratio; NS, not significant; PLT, platelets; PTTr, partial thromboplastin time ratio; Q_B_, blood flow; Q_REP_, replacement solution flow (pre/post, pre-filter/post-filter re-infusion); Q_DIAL_, dialysate flow; UF, ultrafiltration.
